# A Potential Role for the Inhibition of PI3K Signaling in Glioblastoma Therapy

**DOI:** 10.1371/journal.pone.0131670

**Published:** 2015-06-29

**Authors:** Stephanie Ströbele, Matthias Schneider, Lukas Schneele, Markus D. Siegelin, Lisa Nonnenmacher, Shaoxia Zhou, Georg Karpel-Massle, Mike-Andrew Westhoff, Marc-Eric Halatsch, Klaus-Michael Debatin

**Affiliations:** 1 Department of Pediatrics and Adolescent Medicine, University Medical Center Ulm, Ulm, Germany; 2 Department of Neurosurgery, University Medical Center Ulm, Ulm, Germany; 3 Department of Pathology and Cell Biology, Columbia University Medical Center, New York, New York, United States of America; 4 Department of Clinical Chemistry, University Medical Center Ulm, Ulm, Germany; University of Navarra, SPAIN

## Abstract

*Glioblastoma multiforme* (GBM) is the most common primary brain tumor and among the most difficult to treat malignancies *per se*. In almost 90% of all GBM alterations in the PI3K/Akt/mTOR have been found, making this survival cascade a promising therapeutic target, particular for combination therapy that combines an apoptosis sensitizer, such as a pharmacological inhibitor of PI3K, with an apoptosis inducer, such as radio- or chemotherapy. However, while *in vitro* data focusing mainly on established cell lines has appeared rather promising, this has not translated well to a clinical setting. In this study, we analyze the effects of the dual kinase inhibitor PI-103, which blocks PI3K and mTOR activity, on three matched pairs of GBM stem cells/differentiated cells. While blocking PI3K-mediated signaling has a profound effect on cellular proliferation, in contrast to data presented on two GBM cell lines (A172 and U87) PI-103 actually counteracts the effect of chemotherapy. While we found no indications for a potential role of the PI3K signaling cascade in differentiation, we saw a clear and strong contribution to cellular motility and, by extension, invasion. While blocking PI3K-mediated signaling concurrently with application of chemotherapy does not appear to be a valid treatment option, pharmacological inhibitors, such as PI-103, nevertheless have an important place in future therapeutic approaches.

## Introduction

*Glioblastoma multiforme* (GBM) is a common primary brain tumor and one of the most lethal cancer, with an average patient's life expectancy of ~12 month post-diagnosis [1]. Despite an intensive multi-modular treatment regime, consisting of surgical resection, radiation and several courses of the chemotherapeutic agent temozolomide (TMZ) [2], therapeutic successes are only rarely achieved. Two key features of GBM are frequently cited as reasons for treatment failure: The malignancies highly invasive nature and it's intrinsic resistance to apoptosis.

While GBM virtually never metastasizes to distant sites, it grows diffusely and highly invasive, infiltrating the surrounding brain tissue and thus making localized treatment, e.g. surgery, particularly ineffective [3]. Crucially, the presence of these invasive GBM cells is sufficient to cause progressive neurological dysfunctions and even death in the absence of a distinct tumor mass [4]. Indeed, it has been repeatedly suggested that GBM should not be viewed as a tumor within the brain, but as a systemic, i.e., whole brain disorder (for example, [5, 6]).

Induction of apoptosis, the dominant mechanism by which most radio- and chemotherapies eliminate cancerous cells, requires induction of cell death pathways which may be counteracted by increased activity of survival signaling cascades [7]. Therefore in recent years the addition of small molecule inhibitors, targeting aberrantly activated survival signaling cascades, to traditional therapeutic regiments was investigated as a promising new approach. This is of particular interest to Glioblastoma, as in 88% of all glioma genetic alterations have been found in the PI3-Kinase/Akt/mTOR network [8, 9], a signaling cascade for which a multitude of pharmacological inhibitors are currently on the market [10]. However, the modulation of the PI3K/Akt/mTOR signaling cascade in an *in vivo* or even clinical setting has been less than promising [11–13].

Interestingly, we and others previously showed that inhibition of PI3K/Akt/mTOR-mediated signaling in Glioblastoma cell lines strongly amplifies cell death induced by radiotherapy and a wide range of chemotherapeutics (for example, [14–20]), suggesting that it should be an ideal candidate for targeted combination therapy, i.e. the pairing of a pharmacological inhibitors of cell signaling (sensitizers)–such as the PI3K/mTOR inhibitor PI-103 –with conventional radio- or chemotherapy (inducers). To address this discrepancy found in the literature, the failure of inhibitors of PI3K signaling in a clinical setting versus promising experimental results, we used a different cellular system to investigate the effects of PI3K inhibition on GBM cells. Instead of using established cell lines we used three matched pairs of cells derived directly from patient material, either cultured under cell culture conditions optimized for stem cells (SC), or short-term differentiated into primary cells (DC).

## Material and Methods

### Primary cultures of GBM

Primary GBM cells were isolated by mechanical disaggregation from surgical specimens obtained from three patients with WHO IV glioma (G35, G38 and G40) as described previously [21]. The stem cell-like phenotype was maintained by culturing cells as free-flowing spheres in DMEM/F-12 (HAM) medium (Gibco, Life Technologies, Darmstadt, Germany), supplemented with EGF (Biomol GmbH, Hamburg, Germany), bFGF (Miltenyi Biotec GmbH, Bergisch Gladbach, Germany) and B27 (Gibco, Life Technologies). Cells were differentiated by allowing them to adhere in the presence of DMEM (Gibco Life Technologies), supplemented with 10% FCS (Biochrom, Berlin, Germany) and penicillin/streptomycin (Biochrom). Differentiated cell populations were maintained for less than 10 weeks [22].

The study was approved by the Ethics Committee, Medical Faculty, Ulm University.

### Cell lines

U87 and A172 cell lines were obtained from ATCC (Manassas, VA, USA) and maintained in DMEM (Gibco Life Technologies), supplemented with 10% FCS (Biochrom), glutamine (Biochrom) and penicillin/streptomycin (Biochrom).

### Inhibitors and drugs

Temozolomide (Sigma-Aldrich, Hamburg, Germany)

Irinotecan (Tocris, Wiesbaden, Germany)

PI-103 (Cayman Chemical, Mi, USA)

### Viability assay

The readout for viability was metabolic activity, which was assessed by an MTT assay as previously described [23]. Briefly, the terazolium dye 3-(4,5-dimethylthiazol-2-yl)-2,5-diphenyltetrazolium bromide (MTT) is reduced by viable cells to a colored, water-insoluble formazan. Therefore, for the final phase of incubation (approximately three hours) growth medium was replaced by Phenol red-free medium containing 1mg/ml MTT salt (Carl Roth, Karlsruhe, Germany). The reaction was stopped by addition of isopopanol for 0.5 hour. This was followed by analysis with a microplate reader at λ: 550;630 (EL800 BioTek, Bad Friedrichshall, Germany).

### Changes in cell number

Cells were seeded and allowed to proliferate for indicated times. This was followed by either prolonged treatment with a trypsin/EDTA solution (Biochrom) until all cells were in suspension (DCs), or prolonged mechanical dissociation until cell spheres were dispersed (SCs). The cell suspension was diluted 1:100 in CASYton solution (Innovatis, Reutlingen, Germany) and cell numbers were determined using CASY1 DT (Innovatis).

### Apoptosis measurement

The apoptosis readout was DNA fragmentation as assessed by fluorescence-activated cell-sorting (FACScan, Becton Dickinson, Heidelberg, Germany) analysis of propidium iodide-stained nuclei as previously described [24].

### Protein immunoblotting

Western blot analysis was performed as previously described [25]. Briefly, after lysis and determination of protein concentration, samples were separated on a 12% SDS-polyacrylamide electrophoresis gel and transferred onto a Hybond ECL nitrocellulose membrane (Amersham Biosciences, Freiburg, Germany). Proteins were visualized by ECL western blotting detection reagents (Amersham Biosciences), according to manufacturer's instruction, and following antibodies were used: rabbit anti–phospho-Akt (Ser473) antibody (Cell Signaling), rabbit anti-phosph-Akt (Thr308) antibody (Cell Signaling), mouse anti-Akt antibody (Bioscience, Heidelberg, Germany), rabbit anti–phospho-S6 ribosomal protein (Ser235/236) antibody (Cell Signaling), rabbit anti–S6 ribosomal protein antibody (Cell Signaling), or mouse anti-GAPDH antibody (Cell Signaling) followed by goat-anti-mouse IgG or goat-anti-rabbit IgG conjugated to horseradish peroxidase (Santa Cruz Biotechnology, Heidelberg, Germany).

### Fluorescence microscopy

Cells were prepared according to a previously published protocol [25]. Briefly after fixing the cells in 3.7% para-formaldehyde, cells were permeabilized using 0.5% Triton-X and stained with Nestin (Cell Signaling) and GFAP (Santa Cruz Biotechnology) antibodies, which in turn were visualized using an FITC-labelled secondary antibody (Santa Cruz Biotechnology).

Pictures were taken with an AX70 'Provis' microscope (Olympus, Hamburg, Germany).

### Wound-healing/Scratch assay

The semi-directional movement of cells was assessed by observing their migration from a confluent environment into unoccupied space, as previously described [24]. Briefly, cells were grown to ~70% confluence, followed by scarring the monolayer with a sterile micropipette tip. Cells were either stained 1 hr after incubation with the inhibitor (i.e. directly after scarring), or after 19 hrs incubation with the inhibitor (i.e. 18 hrs after scarring), fixed in 4% PFA and stained with Giemsa.

### Random motility

Random movement of cells was analyzed via time-lapse photography, as previously described [24], by taking a picture every 20 min over a time course of 24 hrs. Relative speed of cells was quantified using ImageJ software (Rasband, W.S., ImageJ, U. S. National Institutes of Health, Bethesda, Maryland, USA, http://imagej.nih.gov/ij/, 1997–2011).

### Population Doubling Time (PDT)

Population Doubling Time was calculated using online freeware (Roth V. 2006 <http://www.doubling-time.com/compute.php>).

### Statistical analysis

Statistical significance was assessed by two-sided Student’s *t*-test.

The expected response to combination treatment was calculated as fractional response to drug A (Fa) + fractional response to drug B (Fb)–(Fa x Fb). Bliss analysis was conducted to detect synergistic (ratio of the actual total response and the expected total response > 1.1), additive (this quotient equaled 0.9 to 1.1), or antagonistic effects (quotient < 0.9).

## Results

### Characterizing the effect of PI-103 on GBM cells

**Fig 1:**
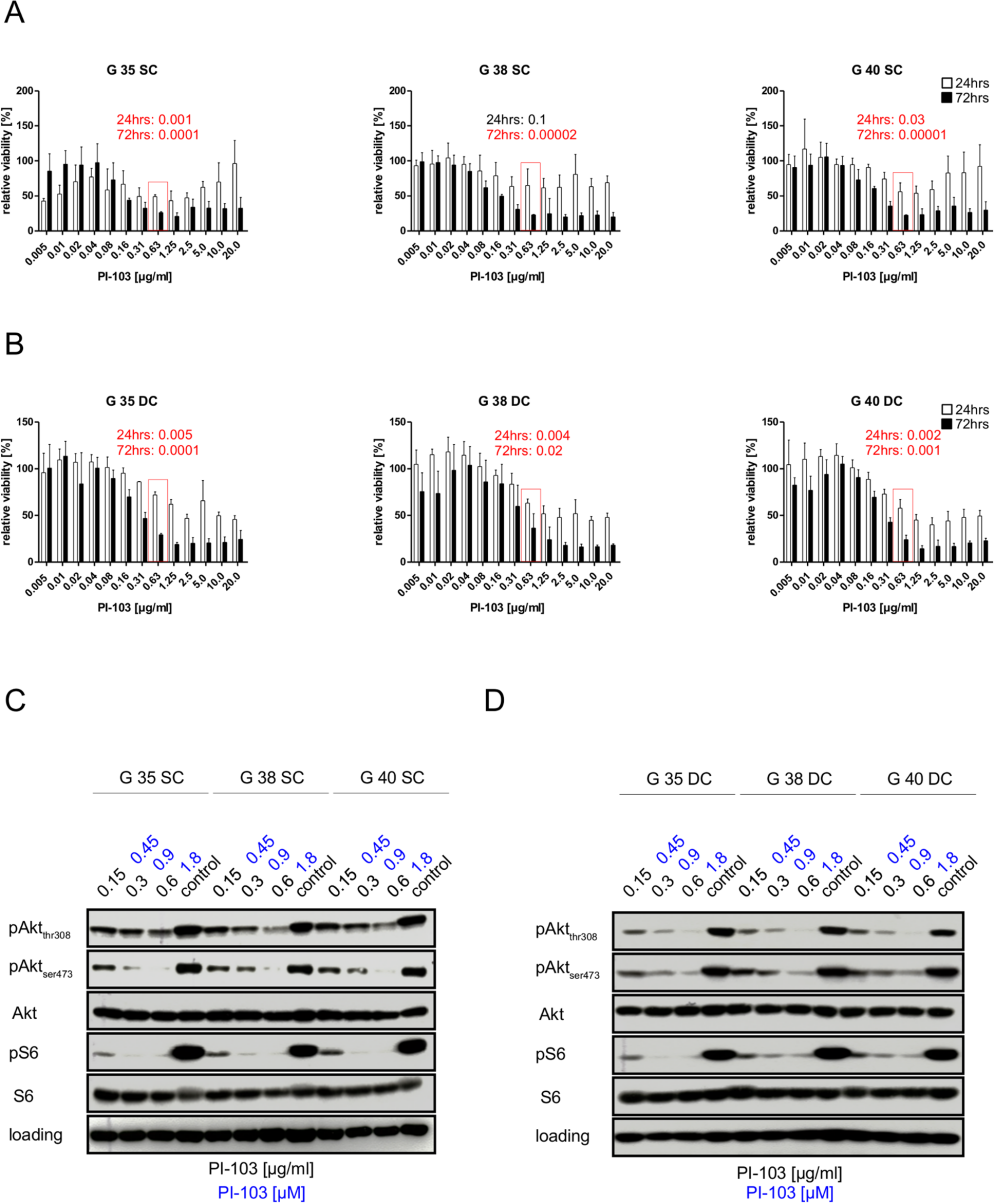
The effects of the PI3K/mTOR inhibitor PI-103 on glioblastoma cells. (A) Different glioblastoma stem cells were either left untreated (i.e. exposed to DMSO solvent alone) or treated for 24 hrs and 72 hrs with indicated concentrations of PI-103, after which the cell population's viability was assessed. Untreated controls were defined as 100%. (B) Distinct differentiated glioblastoma cells (derived from stem cells used in A) were either left untreated (exposed to solvent alone) or treated for 24 hrs and 72 hrs with indicated concentrations of PI-103, after which the cell population's viability was assessed. Untreated controls were defined as 100%. (C) Different glioblastoma stem cells were either left untreated (exposed to solvent alone) or treated for 24 hrs with indicated concentrations of PI-103. Protein expression levels and phosphorylation status of Akt and S6 ribosomal protein served as surrogate read-outs for PI3K and mTOR activity, respectively, and were analyzed by Western blotting, GAPDH served as loading control. (D) Distinct differentiated glioblastoma cells were either left untreated (exposed to solvent alone) or treated for 24 hrs with indicated concentrations of PI-103. Protein expression levels and phosphorylation status of Akt and S6 ribosomal protein served as surrogate read-outs for PI3K and mTOR activity, respectively, and were analyzed by Western blotting, GAPDH served as loading control. Shown in A and B is the mean+SD of at least three independent experiments carried out in triplicate, while in C and D a representative result of two independent experiments is depicted. Red numbers indicate the p-value derived from a two-sided Student's *t*-test.

**Fig 2:**
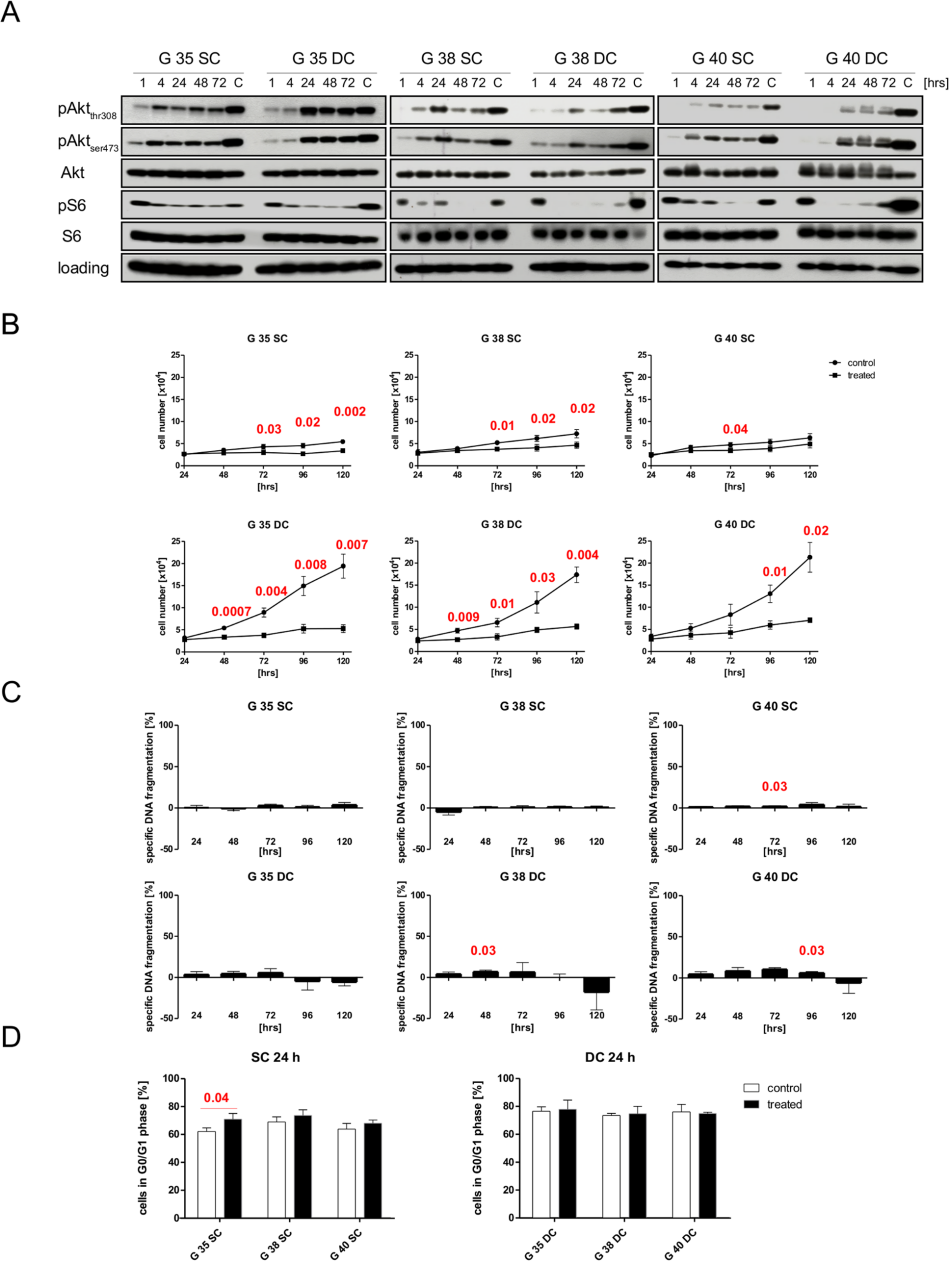
The effects of prolonged exposure to 1.8 μM PI-103 on glioblastoma cells. (A) Different glioblastoma cells, either stem cells (left) or differentiated cells (right) were left untreated (i.e. exposed to DMSO solvent alone) or treated for indicated times with 1.8 μM PI-103. Protein expression levels and phosphorylation status of Akt and S6 ribosomal protein served as surrogate read-outs for PI3K and mTOR activity, respectively, and were analyzed by Western blotting, GAPDH served as loading control. (B) After seeding cells, either untreated (exposed to solvent alone)or treated with 1.8 μM PI-103 were counted every 24 hrs for a total of 120 hrs. (C) Cells were cultured either in the presence or absence of 1.8 μM PI-103 for indicated times, while controls were exposed to the solvent only (DMSO). This was followed by FACS analysis of the DNA fragmentation of propidium iodide-stained nuclei. Treatment induced DNA fragmentation, a surrogate for apoptosis induction, is shown relative to spontaneous cell death of untreated cells. (D) Cells were cultured either in the presence or absence of 1.8 μM PI-103 for 24 hrs, while controls were exposed to the solvent only (DMSO), and the percentage of cells within the live population that reside in the G_0/1_ phase of the cell cycle were determined by FACS analysis of propidium iodide-stained nuclei. Shown in A is a representative result of two independent experiments, while B, C and D depict the mean+SD of three independent experiments carried out in triplicate. Red numbers indicate the p-value derived from a two-sided Student's *t*-test.

First, we characterized the effect of the dual kinase inhibitor PI-103, which blocks PI3K and mTOR activity, on three paired sets of SCs and DCs. G35, G38 and G40 stem cells were chosen from our collection of brain tumor stem cells and allowed to differentiate short term (see Materials and Methods section). These three cell populations were obtained, from a 44-year old male, a 75-year old male and a 57-year old female respectively and have been previously characterized [21]. Briefly, all three do not express detectable PTEN or *O*6-alkylguanine DNA alkyltransferase (MGMT) protein and exhibit high activity of the PI3K signaling cascade, as assessed by AKT and S6 phosphorylation [21]. To find an effective concentration of the inhibitor we monitored metabolic activity for 24 and 72 hrs (Fig 1A and 1B). In SCs the maximal effect of the inhibitor was achieved at a concentration of ~0.6 μg/ml, after which the metabolic activity either increased again or plateaued (Fig 1A), in contrast in DCs metabolic activity did not increase again at higher concentrations and generally slightly higher concentrations were needed to elicit the maximal effect (Fig 1B). As 0.6 μg/ml PI-103 corresponds to 1.8 μM, a concentration in the upper range of commonly used concentrations of this pharmacological inhibitor [26–28], we decided to further concentrate on this concentration, as well as 0.3 μg/ml (0.9 μM) and 0.15 μg/ml (0.45 μM), the latter being the minimum concentration suggested by the manufacturer (Cayman Chemical). Investigating the effect of PI-103 at the level of protein signaling we used phosphorylation of Akt and S6 as surrogate read-outs for the activity of PI3K-mediated signaling 24 hrs after addition of inhibitor. Both, 0.9 and 1.8 μM of PI-103, block S6 phosphorylation almost completely, while the higher concentration has a more potent effect on Akt phosphorylation after 24 hrs (Fig 1C and 1D). The two major phosphorylation sites on Akt, Thr308 and Ser473, appear to be differently affected by the inhibitor, depending on whether SCs or DCs are treated. In SCs phosphorylation of Ser473 seems primarily affected by PI-103 (Fig 1C), while in DCs Thr308 phosphorylation is more sensitive to the inhibitor (Fig 1D). 1.8 μM of PI-103 lead to a rapid and prolonged inhibition of the PI3K/Akt signaling cascade (Fig 2A) that lasts for at least 72 hrs, however the maximal inhibition (as judged by Akt phosphorylation) in SCs lasts for less than 4 hrs (Fig 2A left), while in PCs it is clearly more than 4 hrs (Fig 2A, right). Interestingly, the time kinetic shows a similar bi-phasic effect in the SCs as the dose response kinetic (compare Figs 1A and 2A).

Taken together this data suggest that the regulation of PI3K-mediated signaling, and therefore by extension the role of this signaling cascade, might differ considerably between SCs and DCs. Next we investigated the effects of PI-103 on cellular behavior; 1.8 μM PI-103 profoundly affected the cell number of treated cells, almost completely blocking any increase of cell numbers over a time span of 72 hrs and severely retarding it for at least 120 hrs (Fig 2B). This effect is not due to cytotoxicity, as indicated by the absence of strong, prolonged DNA fragmentation (Fig 2C), but most likely mediated by a block in proliferation, which in PI-103-treated GBM does not seem to be caused by a block in the G_1_/G_0_ phase of the cell cycle (Fig 2D). Interestingly, while 0.9 μM PI-103 also inhibits PI-103 signaling over over a time span of 72 hrs, it has a less striking effect on cell numbers (S1A and S1B Fig). This effect is also not mediated by apoptotic cell death (S1C Fig).

### Assessing the potential of PI3K inhibition in combination with chemotherapy

**Fig 3:**
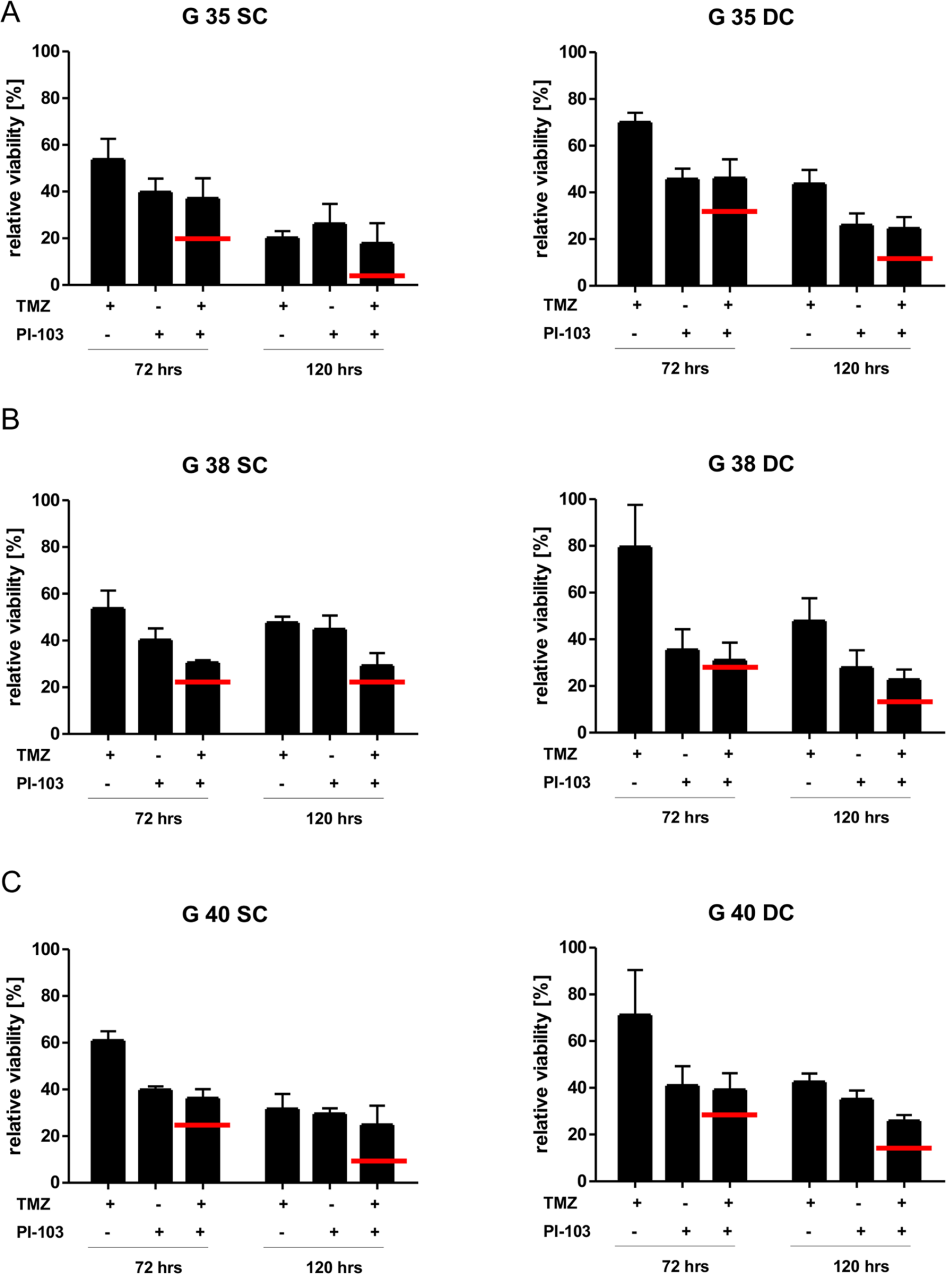
The effects of combination therapy with PI-103 and temozolomide on glioblastoma cell viability. Shown is the relative cell viability of G35 (A), G38 (B) or G40 (C) glioblastoma stem (left) and differentiated (right) cells after treatment with a combination of 1.8 μM PI-103 and 100 μM temozolomide (TMZ) for the indicated times. Shown are the mean+SD of three independent experiments, each the average of six values. The red bar indicates the statistical value that defines the mean of an additive effect.

**Fig 4:**
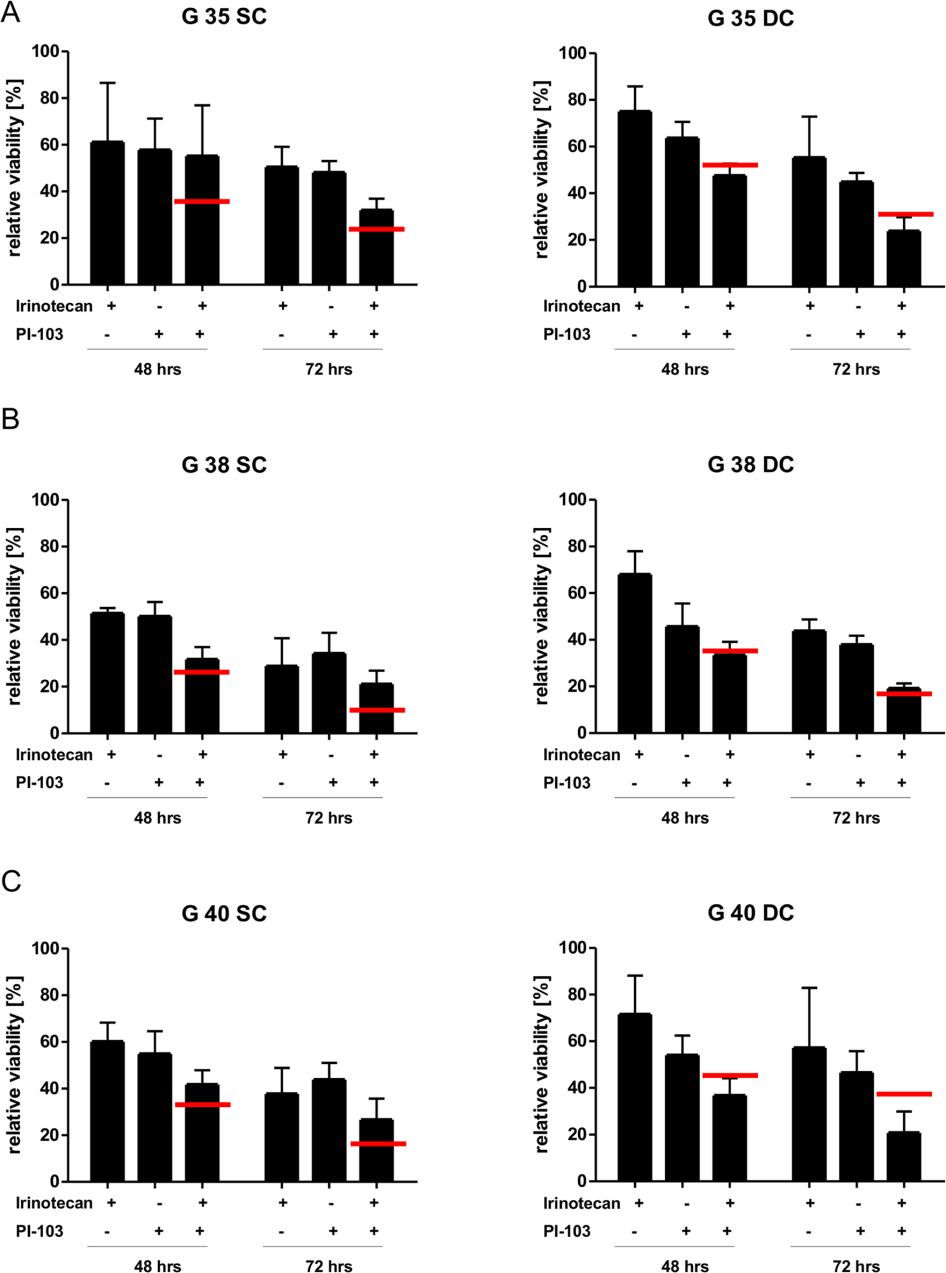
The effects of combination therapy on differentiated glioblastoma cell viability. Shown is the relative cell viability of G35 (A), G38 (B) or G40 (C) glioblastoma stem (left) and differentiated (right) cells after treatment with a combination of 1.8 μM PI-103 and 10nM irinotecan for the indicated times. Shown are the mean+SD of three independent experiments, each the average of six values. The red bar indicates the statistical value that defines the mean of an additive effect.

**Fig 5:**
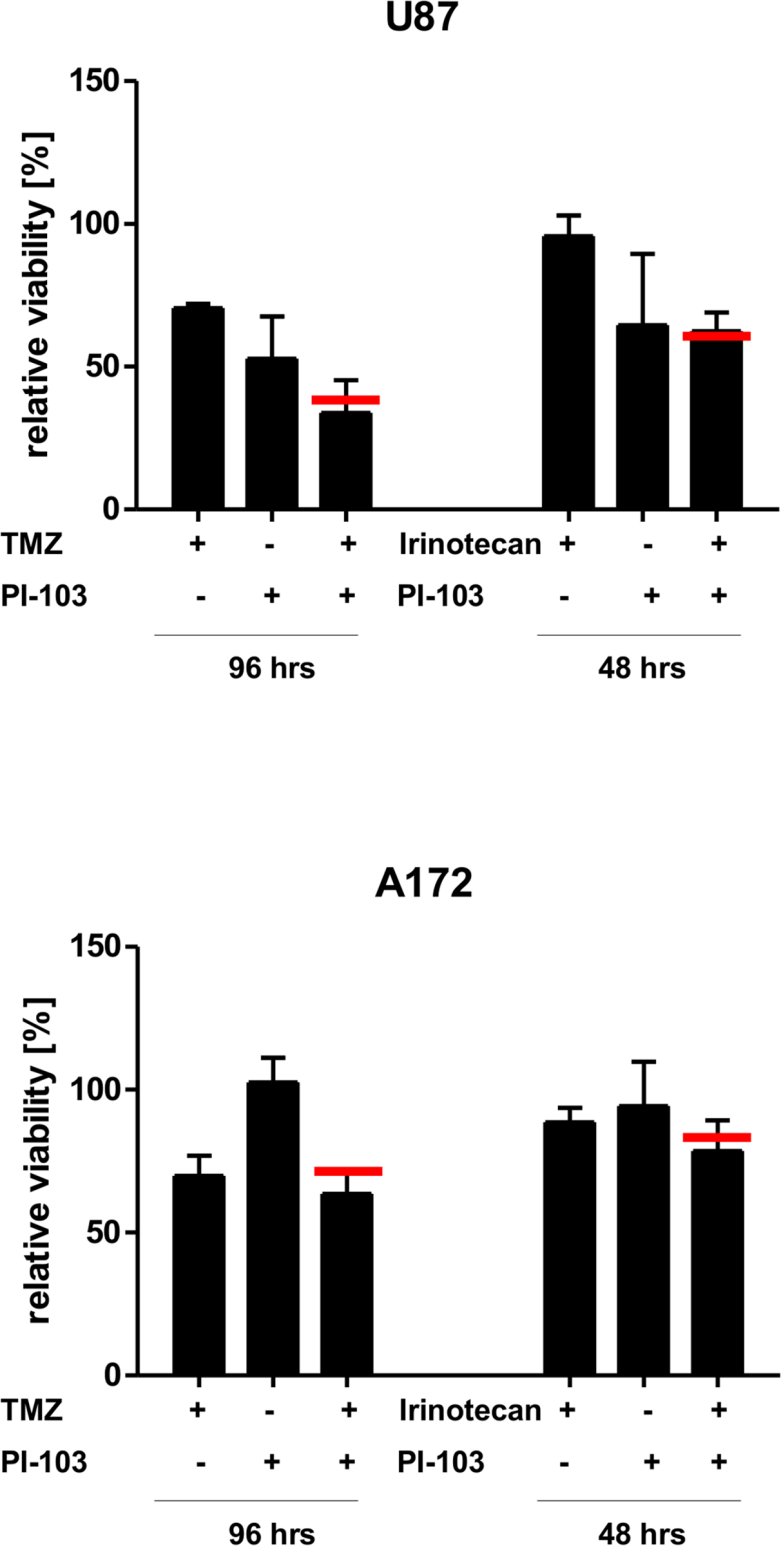
The effects of combination therapy on established glioblastoma cell lines. Shown is the relative cell viability of established glioblastoma cell lines U87MG (upper panel) and A172 (lower pannel) after treatment with a combination of 1.8 μM PI-103 and either 100 μM temozolomide (TMZ) (left) or 10nM irinotecan (right) for the indicated times. Shown are the mean+SD of three independent experiments carried out in triplicate. The red bar indicates the statistical value that defines the mean of an additive effect.

While we have previously shown that pharmacological inhibitors of the PI3K signaling cascade in general, and PI-103 specifically, can be used with chemotherapy to sensitize GBM cells for apoptosis [14–17], this work was mainly done with established cell lines. Therefore, we wanted to verify our results in a more relevant cellular system and investigate potential differences between SCs and DCs, and initially chose TMZ for this set of experiments. Although the synergistic effects of TMZ and PI-103 are relatively weak even in cell lines [15], this chemotherapeutic agent remains the treatment of choice for GBM patients [2]. After establishing a concentration of TMZ and irinotecan which as single treatment did not affect cells fitness more than 50% (S2A and S2B Fig, respectively), we combined 100 μM TMZ with 1.8 μM PI-103 (Fig 3). Interestingly, while the slower proliferating (compare Fig 2B upper row versus lower row) SCs are more sensitive to TMZ treatment alone than DCs (S2A Fig), TMZ with 1.8 μM PI-103 was an antagonistic combination in both, SCs and DCs (Fig 3). As it has been suggested that TMZ is an alkylating agent that damages DNA by generating *O*6-alkylguanine [29], a process which requires cell proliferation, we therefore repeated the experiments with a second chemotherapeutic agent, irinotecan, which causes cell death by topoisomerase inhibition [30] and has shown some promise in GBM treatment [21]. Intriguingly, the use of a second chemotherapeutic agent, combined with 1.8 μM PI-103, did not lead to strong positive effects (i.e. no additive or synergistic effect was observed) in SC cells, but did cause synergism in DC cells (Fig 4), as summarized in Tables 1 and 2. This is stark contrast to cell lines treated under the same conditions, where all observed effects are at least additive (Fig 5, Tables 1 and 2). While the experiments described in Figs 3 to 5 assess the cell viability of the whole cell population over time, i.e. the combination of cell death, proliferation and metabolic activity, we also looked at the induction of apoptosis, the primary mode of cell death by which most chemotherapeutic agents kill cancer cells, to ascertain whether a combination of PI-103 and chemotherapy increases the percentage of cells killed compared to expose to TMZ or irinotecan alone (S3 Fig). While we found striking differences in the apoptosis sensitivity of the various cell populations when exposed to TMZ–as previously suggested SC cells are more resistant than DC cells [31]–we found no statistical significant increase in cell death when comparing combination therapy with chemotherapy alone (S3 Fig). Importantly, the lack of synergism observed is not due to the growth inhibitory effects of PI-103, as no cooperation between drugs and inhibitor could be observed at a concentration of 0.9 μM PI-103, which has a much reduced effect on cell number changes (S2 and S4 Figs). Intrigued, we used a second inhibitor, U0126, which blocks MEK, as the MEK/ERK signaling cascade also plays an important role in GBM [32], where its activity is often intrinsically linked to PI3K-mediated signaling (e.g. [33–36]). We selected the commonly accepted concentration of 50 *μ*M [37–39] which we ourselves have utilized in the past [40]. Here also the inhibitor had a pronounced effect on cell numbers (S5A Fig), while exhibiting little toxicity (S5B Fig), but combination treatment with either TMZ or irinotecan had very little therapeutically positive effect on cell viability (S5C and S5D Fig).

**Table 1:** The effects of combination therapy on cell viability of glioblastoma cells.

		SC			DC			
		G35	G38	G40	G35	G38		G40
PI-103 + TMZ	72 h	-*	**-**	**-**	**-**		**o**	**-**
	120 h	**-**	**-**	**-**	**-**		**-**	**-**
PI-103 + irinotecan	48 h	**-**	**-**	**-**	**+**		**o**	**+**
	72 h	**-**	**-**	**-**	**+**		**-**	**+**

*) the effects on cell viability, as shown in Figs 3 and 4, classified as (-) antagonistic, (o) additive or (+) synergistic.

**Table 2:** The effects of combination therapy on cell viability of established glioblastoma cell lines.

		U87	A172
PI-103 + TMZl	96 h	+*	**+**
PI-103 + irinotecan	48 h	**o**	**o**
	72 h	**-**	**o**

*) the effects on cell viability, as shown in Fig 5, classified as (-) antagonistic, (o) additive or (+) synergistic.

Taken together this data set suggests that combining inhibitors of key 'survival' signaling cascades in GBM does not necessarily lead to a therapeutic benefit.

### Investigating the role of PI3K signaling during differentiation

**Fig 6:**
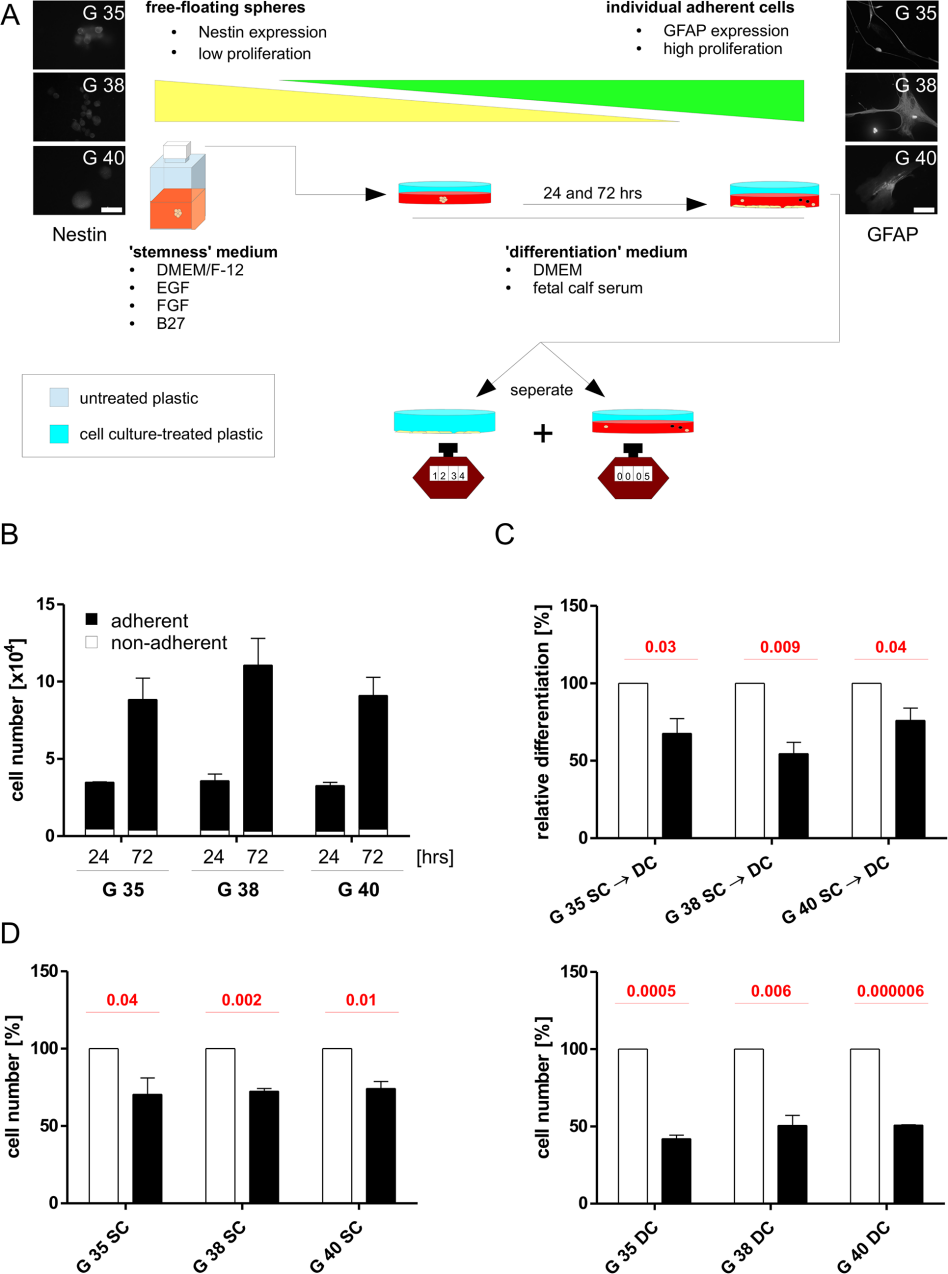
The effects of PI-103 on differentiation. (A) Scheme of the experimental set-up used to collect data presented in B-D, showing Nestin expression in the G 35, G 38 and G 40 stem cells (left) and GFAP expression in the G 35, G 38 and G 40 differentiated cells (right). (B) 24 and 72 hrs after seeding stem cells onto cell culture-treated plastic in the presence of differentiation medium, the total amount of cell, adherent (a surrogate for differentiation) and in suspension (a surrogate for 'stemness' or non-viability) was counted. (C) The relative amounts of differentiated cells (as defined by adhesion) was determined 72 hrs after initiation of differentiation, either in the presence or absence of 1.8 μM PI-103, i.e. controls were exposed to the solvent only (DMSO). (D) The relative amounts of cells, either stem cells (left) or differentiated cells (right) was determined 72 hrs culturing under appropriate conditions, either in the presence or absence of 1.8 μM PI-103, i.e. controls were exposed to the solvent only (DMSO). Shown are the mean+SD of three independent experiments carried out in triplicate. Red numbers indicate the p-value derived from a two-sided Student's *t*-test.

It has previously been suggested that the PI3K signaling cascade may play a role in GBM differentiation (e.g. [41, 42]). To investigate this claim, we allowed SCs to differentiate into DCs, as described in Fig 6A, in the presence or absence of PI-103. Adhesion, a prerequisite for differentiation, occurs within the first 24 hrs, after which cells proliferate rapidly, while the percentage of cells still in suspension (i.e. either dead or undifferentiated) remains at a constant low (Fig 6B). The addition of PI-103 does not prevent adhesion of cells during differentiation, but leads to markedly reduced numbers of DCs after 72 hrs (Fig 6C), however this can easily be explained by the potent effect PI-103 has on cell proliferation (Fig 6D, Table 3). Indeed the reduction in differentiation is almost equal in the reduction of total cell numbers (compare Fig 6C and 6D), suggesting that under the experimental conditions investigated, no effect on differentiation can be observed. Similarly, inhibition of MEK leads to a reduced number of differentiated cells, which parallels the reduction in proliferation (S5A and S5E Fig). This is an important finding as it clearly indicates that the antiproliferative effect of PI3K inhibition must be taken into account when investigating GBM cell differentiation.

**Table 3:** The relative effects of PI-103 on proliferation and differentiation.

	G35			G38			G40		
	proliferation		differentiation	proliferation		differentiation	proliferation		differentiation
	SC	DC	SC→ DC	SC	DC	SC→ DC	SC	DC	SC→ DC
PI-103	70.55*	42.07	59.48	72.39	50.69	52.4	74.35	50.86	58.95

*) shown in [%] is the effect a 72 hrs treatment with 1.8 μM PI-103 has on either proliferation or differentiation, e.g. 70.55 indicates that after 72 hrs in the presence of PI-103 the amount of G35 SC is reduced to 70.55% compared to 100% untreated control.

Of note: The presumed effect on differentiation never exceeds the effect on proliferation, i.e. can be explained by this effect alone.

### Ascertaining the contribution of PI3K signaling to GBM cell motility

**Fig 7:**
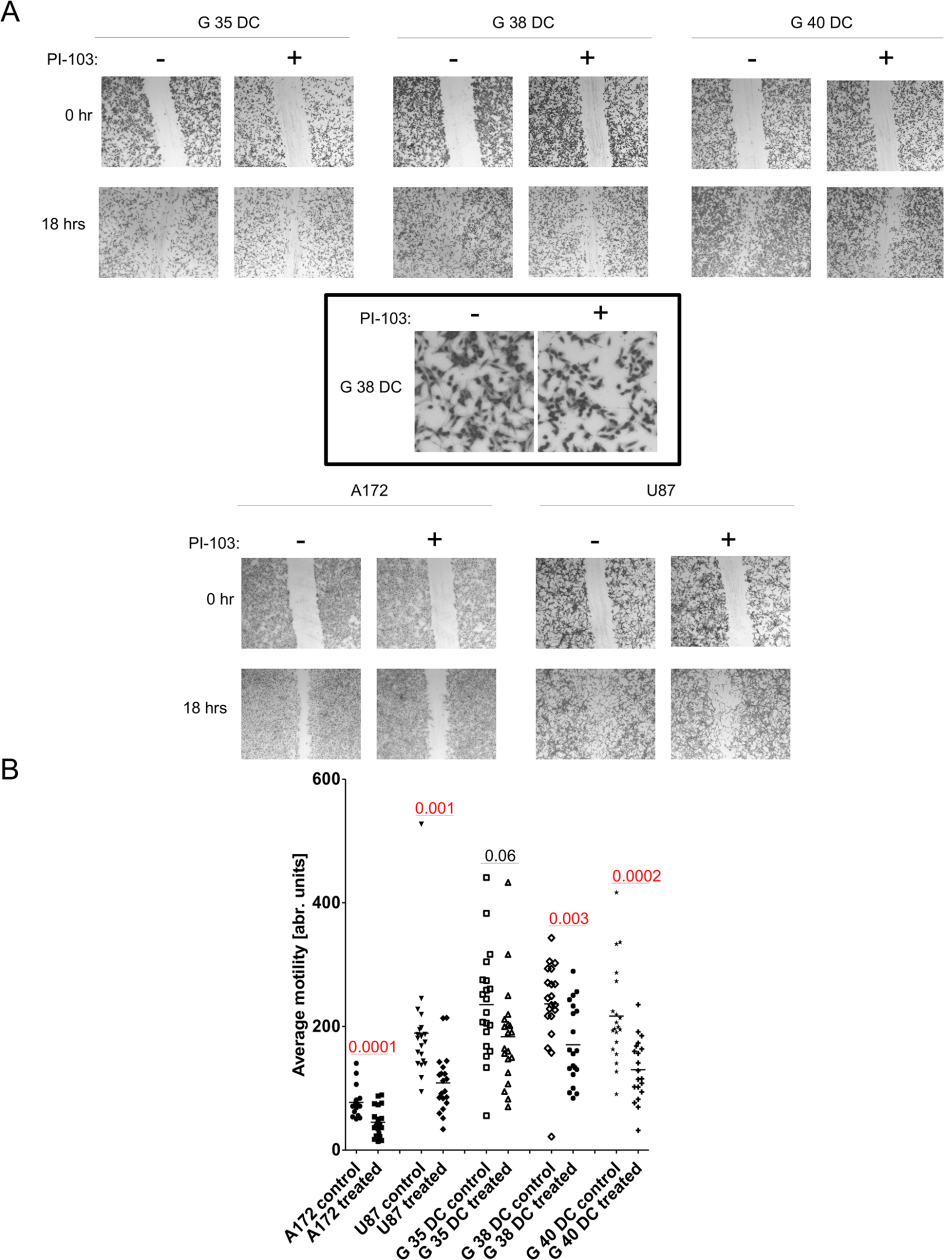
The effects of PI-103 on cellular motility. (A) Differentiated glioblastoma cells (upper panel) or established cell lines (lower panel) were allowed to grow ~70% confluent, upon which they were either left untreated or pretreated with 1.8 μM PI-103 for 1 hr (see box-out for morphological example of 1 hr treatment). Cellular monolayers when then scared with a micropipette tip and the wound was allowed to close for 18 hrs, in the presence or absence of PI-103, i.e. controls were exposed to the solvent only (DMSO). (B) The random motility of glioblastoma cells, both cell lines and freshly differentiated cells, was tracked via timelapse microscopy for 6 hrs in the presence or absence of 1.8 μM PI-103, i.e. controls were exposed to the solvent only (DMSO). Shown in A is a representative result of three independent experiments performed in triplicate, while B depicts the summary of two independent experiments, in each 10 cells were tracked and their average travel distance assessed. Red numbers indicate the p-value derived from a two-sided Student's *t*-test.

Finally, we investigated a potential role of PI3K-mediated signaling to GBM cell motility and, therefore, to the highly motile, invasive phenotype which should be considered a defining feature of GBM and contributes greatly to its resistance to treatment [3]. After pretreatment of a DC monolayer for 1 hr with PI-103, we set a scar and followed its closure for 18 hrs (Fig 7A). The presence of PI-103 clearly led to a retardation, but not a block, in wound healing, in both primary differentiated cells (Fig 6A, upper panel) and established cell lines (Fig 6A, lower panel), this might be due to less cell spreading, as suggested by the example shown in the box-out (Fig 6A). To better quantify this effect, we performed a time lapse analysis, whereby we followed the random motility of cells for 6 hrs (Fig 6B). Although not statistically significant in one comparison, all cells investigated exhibit reduced motility in the presence of PI-103.

This set of experiments clearly indicates a potential role for PI3k-mediated signaling in GBM cell motility and, by extension, invasion. As the invasive nature of GBM makes it one of the hardest to treat tumors, these findings are of great therapeutic relevance.

## Discussion

In this study we have used three previously described [21] primary patient-derived GBM stem cells (SCs) and compared the effect of PI3K inhibition via the pharmacological inhibitor PI-103 to that on cells differentiated out of those stem cells (DCs). While the field of cancer stem cells is not without controversy [43], we relied on the currently most accepted approximation of GBM stem cell, i.e. grown as spheres in the absence of serum and positive for Nestin, while negative for GFAP expression [44, 45], as shown in Fig 7. We also observed two additional previously described differences between SC and DC populations which further increase our confidence in the chosen model: 1. As previously described by others [46], we also found the SCs to proliferate significantly slower than their differentiated counterparts (Table 4). 2. In accordance with previously published findings [31], SCs are more resistant to TMZ-induced apoptosis than DCs (S3 Fig), which has been suggested to be a direct consequence of the reduced SC proliferation [46]. Interestingly, we did not see this reflected in apoptosis rates upon exposure to irinotecan, further supporting Mitchison's contention that proliferation and apoptosis indexes do not exist in a mere linear relationship [47].

**Table 4:** The effect of 1.8 μM PI-103 on the population doubling times (PDT).

	G 35				G 38				G 40			
	SC		DC		SC		DC		SC		DC	
	untreated	treated	untreated	treated	untreated	treated	untreated	treated	untreated	treated	untreated	treated
PDT	84.68*	197.38	39.93	114.82	67.72	108.08	41.1	99.26	71.63	97.07	38.57	83.22

*) the population doubling time was calculated based on the data shown in Fig 2B, under consideration that the concentration of PI-103 used does not induce apoptosis (Fig 2C).

Upon exposure to 1.8 μM PI-103 both sets of cell population exhibited a markedly reduced proliferation (Fig 2B, Table 4), without exhibiting an increase in apoptosis (Fig 2C). This finding distinguishes GBM from other tumour entities that show an increased PI3K activity. For example, inhibition of PI3K signalling in Hodgkin lymphoma leads to increased apoptosis induction, even in the absence of additional stress factors [48]. It appears that strong PI3K-mediated signalling is not essential for GBM survival, but rapid proliferation. The addition of external stress, here in the form of two chemotherapeutic agents TMZ and irinotecan (Figs 3 and 4, respectively), did not alter this perception. While inhibitor and drugs individually had a clear effect on the viability (i.e. the combination of cell death, proliferation and metabolic activity) of the investigated cell populations, the combination of both (potential) sensitizer and inducer of apoptosis only occasionally achieved an additive or synergistic effect (Tables 1 and 2). These findings are also reflected when looking at apoptosis directly (S3 Fig), suggesting that using the experimental set-up described here, inhibition of PI3K is not a promising sensitizer for cell death. While the data presented here is in contrast to several per-clinical studies, including our own [15], which have often been performed with established cell lines [14–17], that only poorly mimic gene and protein expression profiles of GBM tumors [49, 50], it is in line with clinical data [11–13] and suggests that simply combining an inhibitor of the PI3K signaling cascade with chemotherapy to treat GBM is not a recommendable strategy.

However, our previous data mainly focusing on a different tumor entity strongly indicates that the sequential timing of a treatment combination can significantly affect the potential efficacy of that combination, altering it from antagonistic to synergistic [16]. This aspect remains to be clarified in future work. Furthermore, we recently showed that a potent complex combination therapy used in the clinic, loses its *in vivo* potency when rapamycine, a relatively downstream inhibitor of the PI3K signaling cascade, is replaced with GDC-0941, a more upstream inhibitor, clearly indicating that carefully applied inhibition of certain aspects of the PI3K signaling cascade can and will have a crucial place in future GBM treatment strategies [21].

Furthermore, while the focus of therapies generally lies on tumor-specific cytotoxicity, it should be pointed out that the cytostasis induced by this monotreatment with PI-103 can be considered a valuable contribution to GBM therapy [51]. Further experiments should determine whether long term cytostasis induced by PI3K/mTOR inhibition finally leads to cell death [51], or can be used to chronify the malignancy [7], both potentially interesting therapeutic avenues to explore in GBM treatment, in particular considering the anti-invasive effect of PI-103 discussed further below.

This cytostatic effect of PI3K/mTOR inhibition has been previously described for both GBM and other tumor entities [28, 48, 52, 53] and is often cited as *caveat* for the use of PI3K inhibitors in combination therapy with classical chemotherapy. While it has been suggested that anti-mitotic drugs exert a more potent effect on highly proliferating cells, this is almost certainly an over-generalization [47]. It should be pointed out that although in both viability and cell death assay the addition of PI-103 frequently failed to synergize with the effects of the chemotherapeutic agents, the addition of PI-103 to either drug did not significantly reduce the effect of the latter compared to drug treatment alone. In experiments with GBM cell lines the anti-proliferative effect of PI3K inhibition did not prevent PI3K inhibition to sensitize the cells for several chemotherapeutic drugs, as well as radiotherapy [14–20]. We also saw this, when exposing the established cell lines A172 and U87, which are among the panel of GBM cell lines characterized by Ishii and co-workers and also express no wild-type PTEN [54], to either irinotecan or TMZ. Therefore, it does not seem unreasonable to consider PI3K inhibition as part of a GBM treatment protocol, not in terms of increasing the efficacy of chemo- and/or radiotherapy, but in order to keep the disease more stable. This is of particular interest, if one considers additional roles that the PI3K signaling cascade might have in GBM, for example in differentiation and invasion.

Over a decade ago differentiation therapy, i.e. the reduction of SCs in solid tumors by treatment with agents that induce differentiation [55], has been proposed as a novel therapeutic approach. In GBM PTEN has been putatively identified to contribute to differentiation [56, 42], however, in contrast to some recent publications [41, 42, 57, 58], several of which focus on established cell lines [57, 58], we find no evidence of PI3K signaling contributing to the differentiation from SC to DC, except in a trivial sense. Alterations in the number of DCs (defined morphologically and by GFAP expression) that can be induced from SCs in the presence of absence of PI-103 are most likely due to the sharp decrease in proliferation upon PI3K inhibition and not a direct effect of an altered differentiation potential. Several of the aforementioned publications do not address this possibility.

Finally, we do see a strong contribution of PI3K signaling to cellular motility and therefore GBM invasion. As the spread of GBM throughout the brain is one of the defining features of this malignancy [5, 6] and makes full surgical resection and effective localized radiotherapy almost impossible to achieve, these findings are of particular importance. A recent review which discusses the role of PI3K inhibition in GBM and neuroblastoma therapy, highlights the possibility that PI3K/Akt facilitate the invasive phenotype of GBM, both in terms of motility and survival under stress [59]. As standard therapy has been shown to increase invasion in the surviving fraction of GBM cells [60], this finding suggests a potential key role for PI3K inhibition in GBM therapy by increasing the therapeutic window via two distinct routes inhibition of rapid proliferation and blockage of cell migration.

In summary, inhibition of PI3K signaling in GBM can have a potent anti-proliferative and anti-invasive effect and should therefore be considered a promising addition to our therapeutic arsenal. The remaining challenge is having to combine pharmacological inhibitors with current treatment schedules as to avoid antagonistic interactions. Importantly, the use of established cell lines to answer this questions is not advisable, as they poorly mimic the effects of combination therapy observed in SCs and DCs.

## Supporting Information

S1 Fig The effects of prolonged exposure to 0.9 μM PI-103 on GBM cells. (A) Different GBM cells, either stem cells (left) or differentiated cells (right) were left untreated (i.e. exposed to DMSO solvent only) or treated for indicated times with 0.9 μM PI-103. Protein expression levels and phosphorylation status of Akt and S6 ribosomal protein served as surrogate read-outs for PI3K and mTOR activity, respectively, and were analyzed by Western blotting, GAPDH served as loading control. (B) After seeding cells, either untreated (exposed to solvent only) or treated with 0.9 μM PI-103 were counted every 24 hrs for a total of 120 hrs. (C) Cells were cultured either in the presence or absence of 0.9 μM PI-103 for indicated times, followed by FACS analysis of the DNA fragmentation of propidium iodide-stained nuclei. Treatment induced DNA fragmentation, a surrogate for apoptosis induction, is shown relative to spontaneous cell death of untreated cells. Shown in A is a representative result of two independent experiments, while B and C depict the mean+SD of three independent experiments carried out in triplicate. Red numbers indicate the p-value derived from a two-sided Student's *t*-test.
(TIF)

S2 Fig The effects of chemotherapeutic agents on GBM cell viability. (A) Shown is the relative cell viability of G35, G38 and G40 GBM cells, stem cells on the left, differentiated cells right, after 72 hrs of treatment with indicated concentrations of temozolomide (TMZ). (B) Shown is the relative cell viability of G35, G38 and G40 GBM cells, stem cells on the left, differentiated cells right, after 48 hrs of treatment with indicated concentrations of irinotecan. Shown are the mean+SD of three independent experiments carried out in triplicate.
(TIF)

S3 Fig Apoptosis induction by combination therapy in GBM cells. G35, G38 and G40 GBM stem cells (A), G35, G38 and G40 GBM differentiated cells (B) as well as U87 and A172 GBM cell lines (C) were treated for 120 hrs with 100 μM temozolomide, 1.8 μM PI-103 and a combination thereof (upper panels) or for 72 hrs with 10nM irinotecan, 1.8 μM PI-103 and a combination thereof (lower panels) followed by FACS analysis of the DNA fragmentation of propidium iodide-stained nuclei. Treatment induced DNA fragmentation, a surrogate for apoptosis induction, is shown relative to spontaneous cell death of untreated cells. Shown are the mean+SD of three independent experiments carried out in triplicate.
(TIF)

S4 Fig The effects of combination therapy on GBM cell viability. Shown is the relative cell viability of G35, G38 or G40 stem (A) or differentiated (B) GBM cells after treatment with a combination of 0.9 μM PI-103 and either 100 μM temozolomide (TMZ) (upper panels) or 10nM irinotecan (lower panels) for the indicated times. Shown are the mean+SD of three independent experiments, each the average of six values. The red bar indicates the statistical value that defines the mean of an additive effect.
(TIF)

S5 Fig A potential role for the inhibition of MEK signaling in GBM therapy. (A) GBM stem cells (SC) or differentiated cells (DC) were either left untreated (i.e. exposed to DMSO solvent only) or treated for 72 hrs with 50μM U0126 (Cell Signaling, Frankfurt, Germany), after which the cell numbers was assessed. Untreated controls were defined as 100%. (B) GBM stem cells (SC) or differentiated cells (DC) were cultured for 72 hrs either in the presence or absence of 50μM U0126, followed by FACS analysis of the DNA fragmentation of propidium iodide-stained nuclei. Treatment induced DNA fragmentation, a surrogate for apoptosis induction, is shown relative to spontaneous cell death of untreated cells. (C) Shown is the relative cell viability of G35, G38 or G40 GBM stem cells after treatment with a combination of 50μM U0126 and either 100 μM temozolomide (TMZ) for 120 hrs (left) or 10nM irinotecan for 72 hrs (right). (D) Shown is the relative cell viability of G35, G38 or G40 differentiated GBM cells after treatment with a combination of 50μM U0126 and either 100 μM temozolomide (TMZ) for 120 hrs (left) or 10nM irinotecan for 72 hrs (right). (E) The relative amounts of differentiated cells (as defined by adhesion) was determined 72 hrs after initiation of differentiation, either in the presence or absence of 50μM U0126. Shown are the mean+SD of at least three independent experiment performed at least in triplicate. Red numbers in A, B and E indicate the p-value derived from a two-sided Student's *t*-test, the red bar in C and D indicates the statistical value that defines the mean of an additive effect.
(TIF)
