# Correction: A Potential Role for the Inhibition of PI3K Signaling in Glioblastoma Therapy

**DOI:** 10.1371/journal.pone.0134770

**Published:** 2015-07-30

**Authors:** The PLOS ONE Staff

The seventh author’s name is spelled incorrectly. The correct name is: Georg Karpel-Massler. The publisher apologizes for this error.

## References

[1] Ströbele S, Schneider M, Schneele L, Siegelin MD, Nonnenmacher L, Zhou S, et al (2015) A Potential Role for the Inhibition of PI3K Signaling in Glioblastoma Therapy. PLoS ONE 10(6): e0131670 doi:10.1371/journal.pone.0131670 26121251 10.1371/journal.pone.0131670 PMC4488267

