# Supplementary figures and images for: A Potential Role for the Inhibition of PI3K Signaling in Glioblastoma Therapy
Source: PLoS One. 2015 Jun 29;10(6):e0131670. doi: 10.1371/journal.pone.0131670 (PMC4488267; doi:10.1371/journal.pone.0131670)

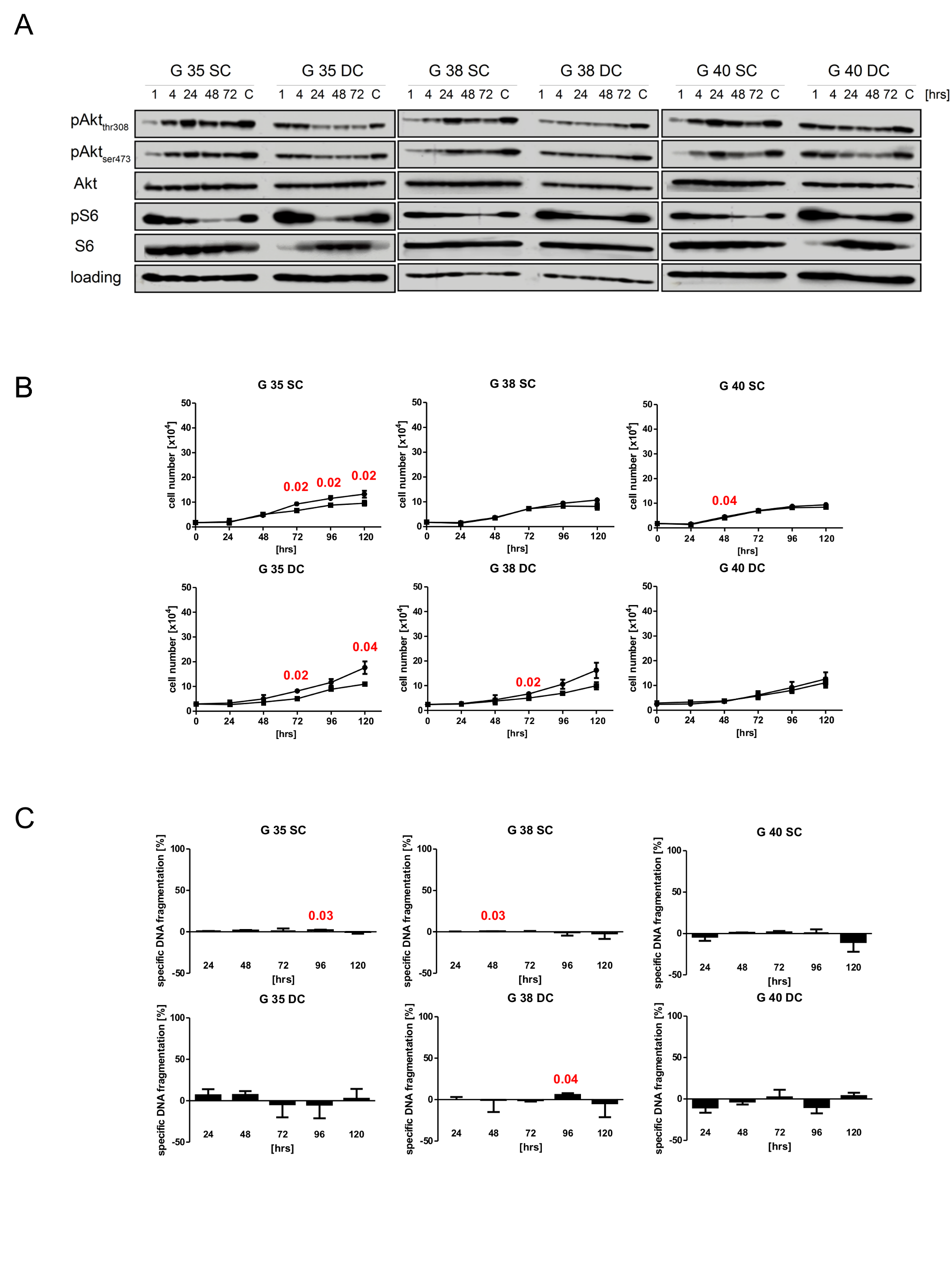

Supplement: S1 Fig — (A) Different GBM cells, either stem cells (left) or differentiated cells (right) were left untreated (i.e. exposed to DMSO solvent only) or treated for indicated times with 0.9 μM PI-103. Protein expression levels and phosphorylation status of Akt and S6 ribosomal protein served as surrogate read-outs for PI3K and mTOR activity, respectively, and were analyzed by Western blotting, GAPDH served as loading control. (B) After seeding cells, either untreated (exposed to solvent only) or treated with 0.9 μM PI-103 were counted every 24 hrs for a total of 120 hrs. (C) Cells were cultured either in the presence or absence of 0.9 μM PI-103 for indicated times, followed by FACS analysis of the DNA fragmentation of propidium iodide-stained nuclei. Treatment induced DNA fragmentation, a surrogate for apoptosis induction, is shown relative to spontaneous cell death of untreated cells. Shown in A is a representative result of two independent experiments, while B and C depict the mean+SD of three independent experiments carried out in triplicate. Red numbers indicate the p-value derived from a two-sided Student's t-test. (TIF) [file pone.0131670.s001.tif]

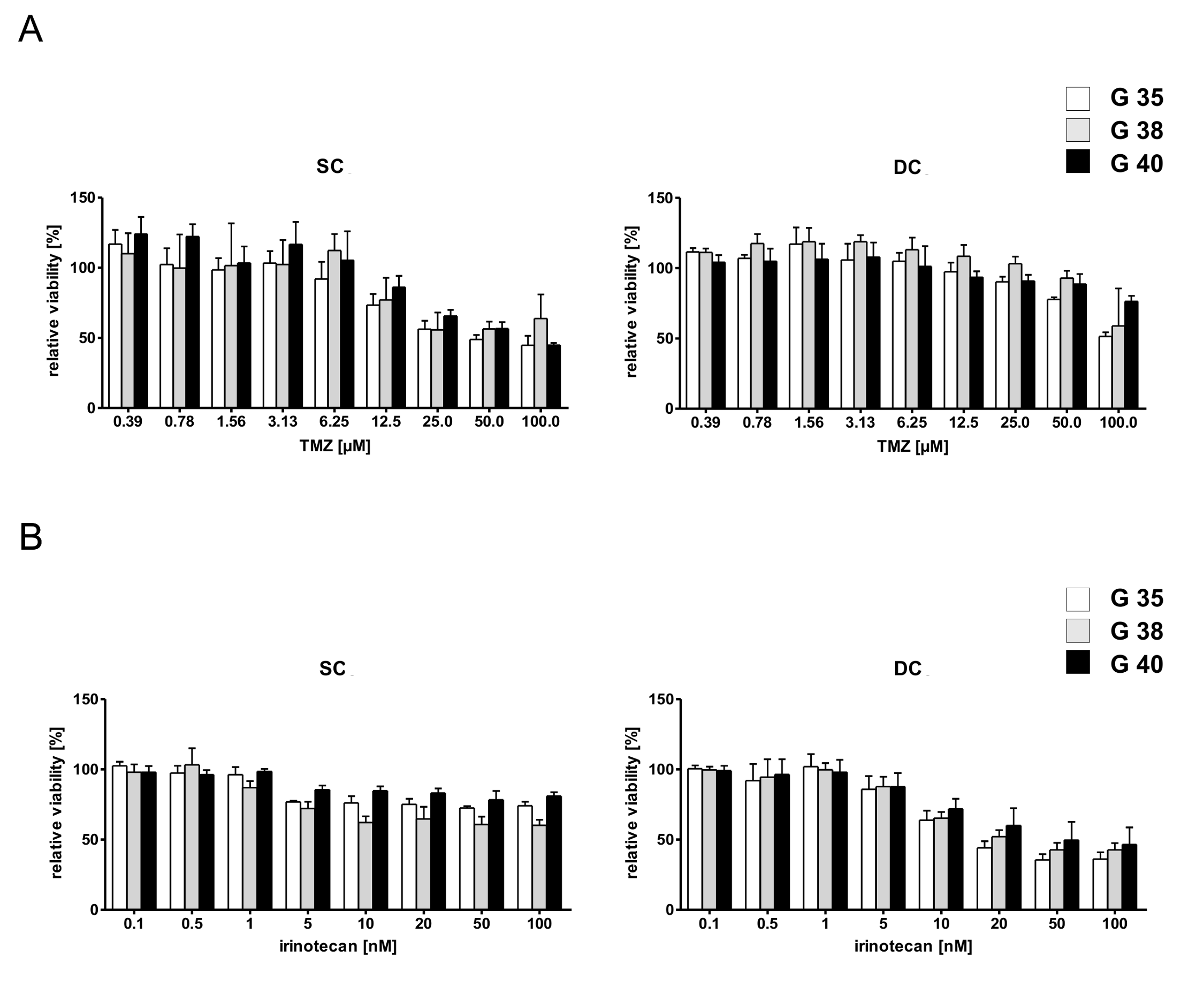

Supplement: S2 Fig — (A) Shown is the relative cell viability of G35, G38 and G40 GBM cells, stem cells on the left, differentiated cells right, after 72 hrs of treatment with indicated concentrations of temozolomide (TMZ). (B) Shown is the relative cell viability of G35, G38 and G40 GBM cells, stem cells on the left, differentiated cells right, after 48 hrs of treatment with indicated concentrations of irinotecan. Shown are the mean+SD of three independent experiments carried out in triplicate. (TIF) [file pone.0131670.s002.tif]

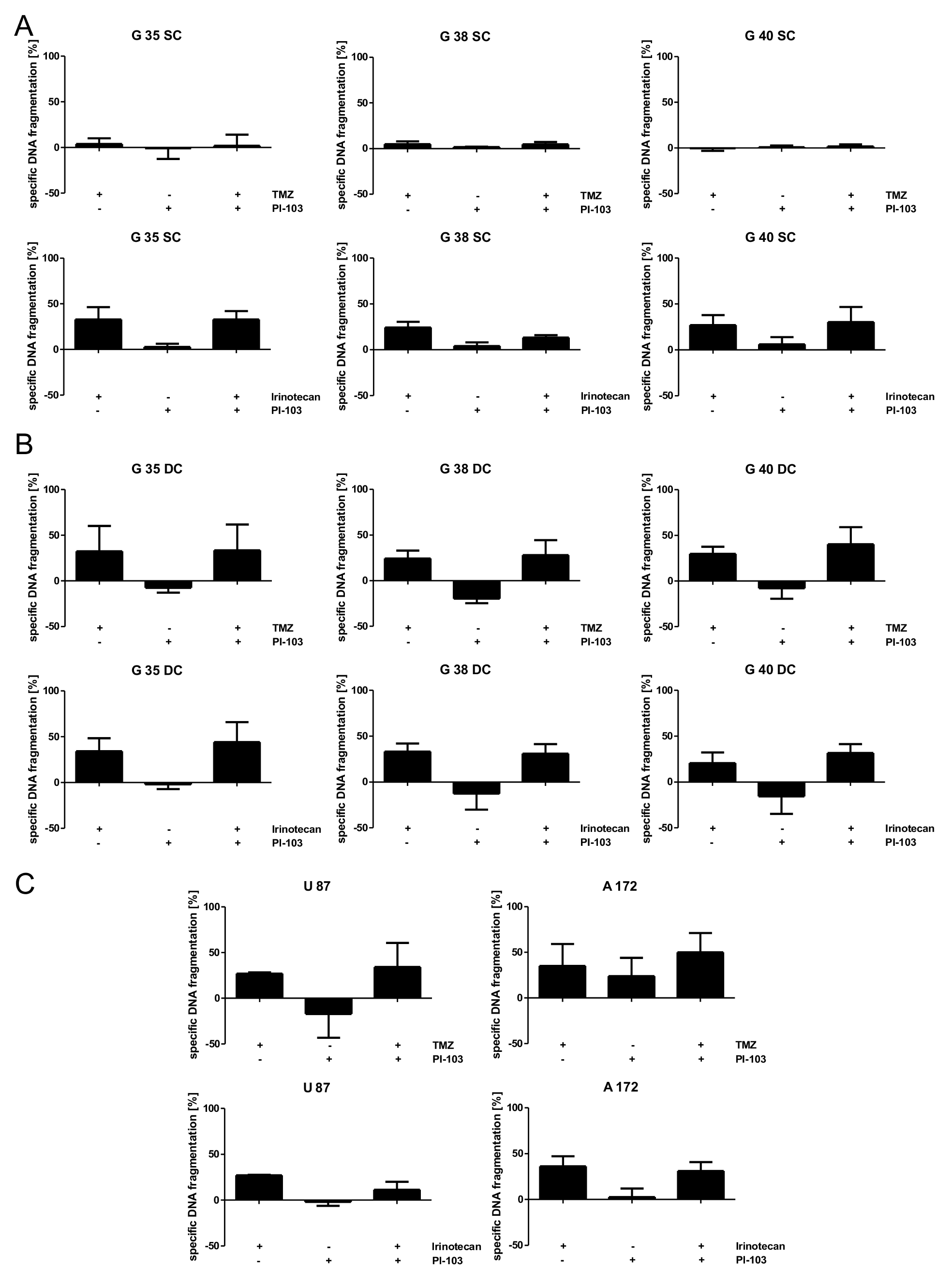

Supplement: S3 Fig — G35, G38 and G40 GBM stem cells (A), G35, G38 and G40 GBM differentiated cells (B) as well as U87 and A172 GBM cell lines (C) were treated for 120 hrs with 100 μM temozolomide, 1.8 μM PI-103 and a combination thereof (upper panels) or for 72 hrs with 10nM irinotecan, 1.8 μM PI-103 and a combination thereof (lower panels) followed by FACS analysis of the DNA fragmentation of propidium iodide-stained nuclei. Treatment induced DNA fragmentation, a surrogate for apoptosis induction, is shown relative to spontaneous cell death of untreated cells. Shown are the mean+SD of three independent experiments carried out in triplicate. (TIF) [file pone.0131670.s003.tif]

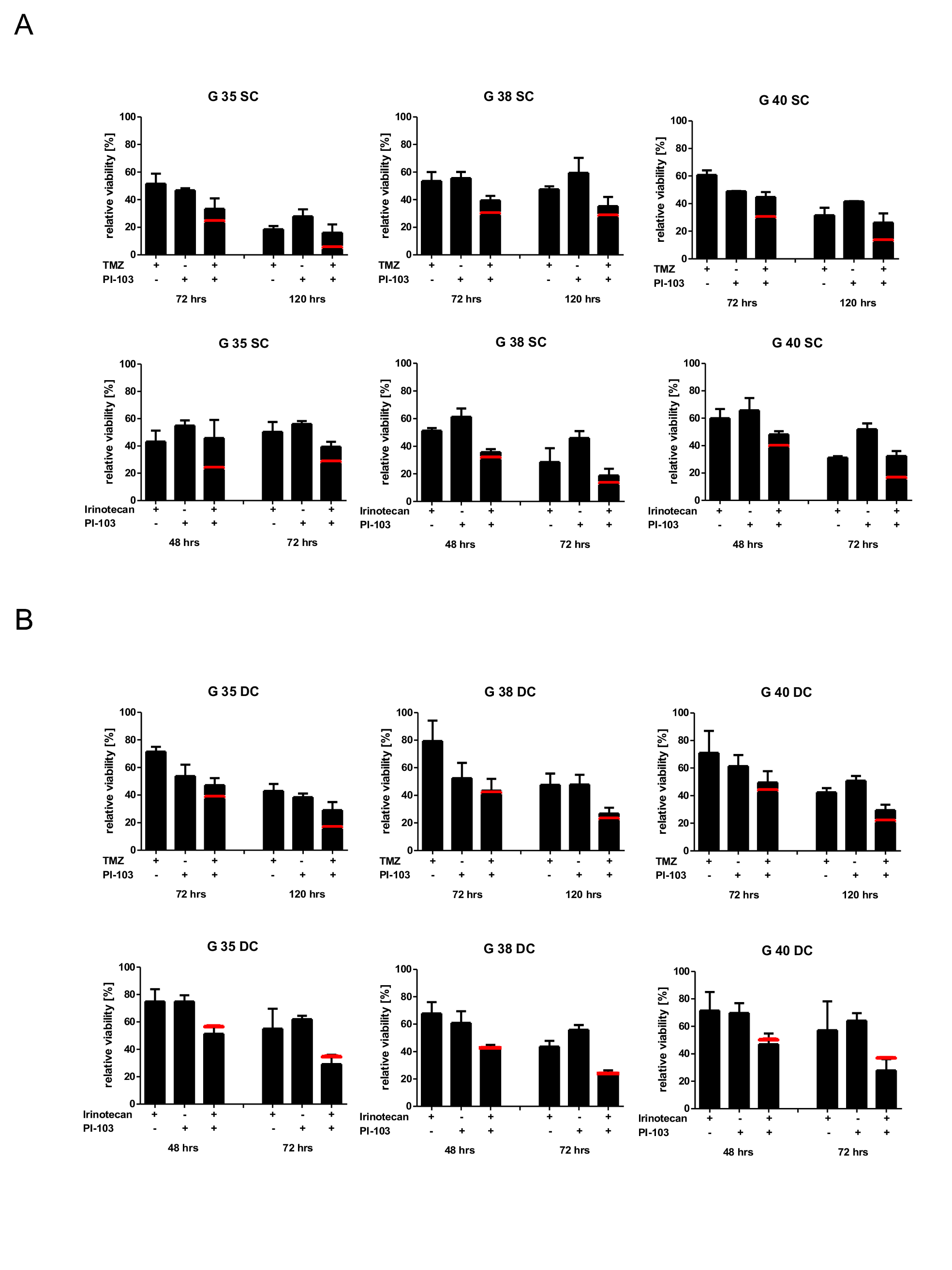

Supplement: S4 Fig — Shown is the relative cell viability of G35, G38 or G40 stem (A) or differentiated (B) GBM cells after treatment with a combination of 0.9 μM PI-103 and either 100 μM temozolomide (TMZ) (upper panels) or 10nM irinotecan (lower panels) for the indicated times. Shown are the mean+SD of three independent experiments, each the average of six values. The red bar indicates the statistical value that defines the mean of an additive effect. (TIF) [file pone.0131670.s004.tif]

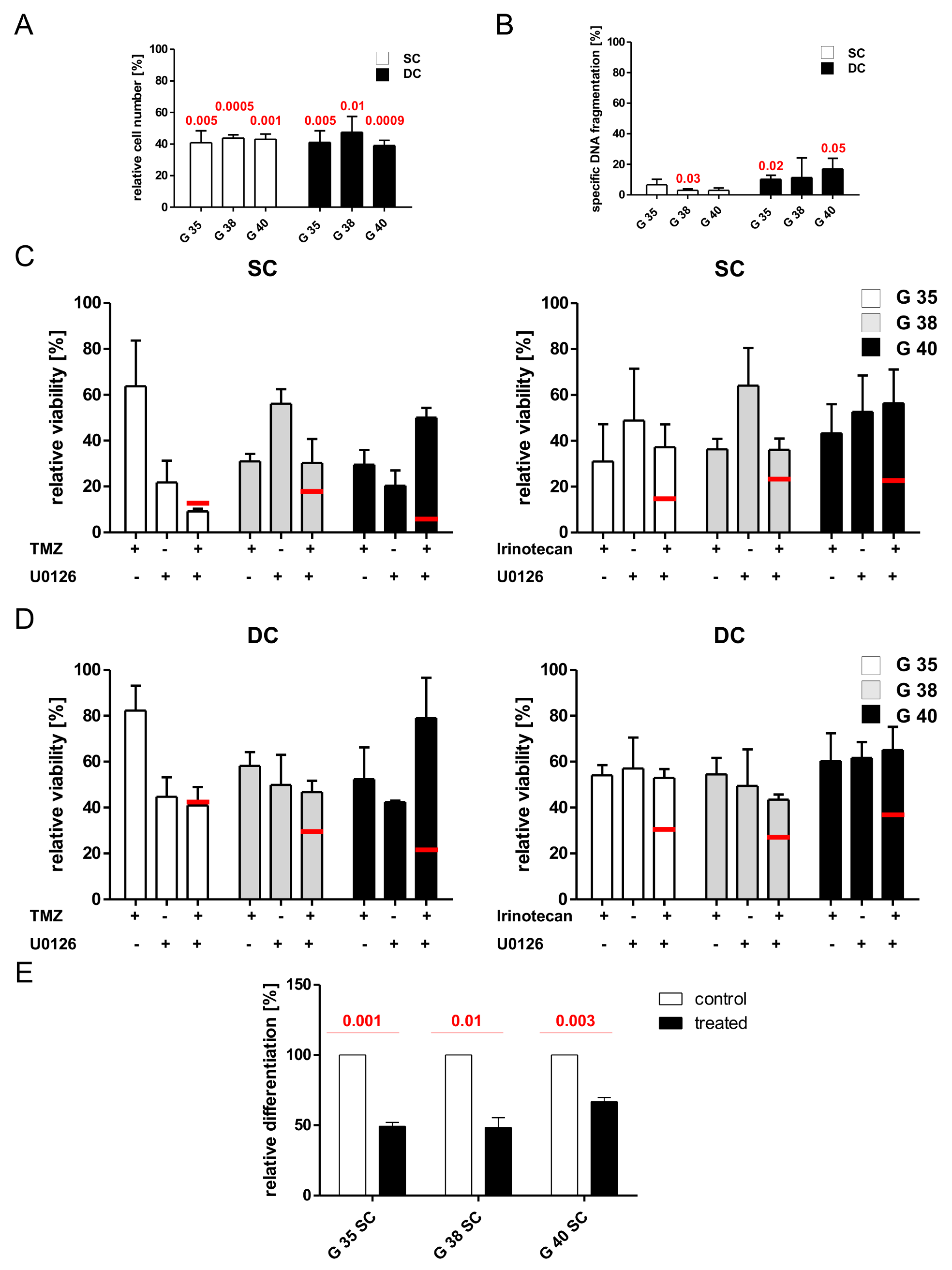

Supplement: S5 Fig — (A) GBM stem cells (SC) or differentiated cells (DC) were either left untreated (i.e. exposed to DMSO solvent only) or treated for 72 hrs with 50μM U0126 (Cell Signaling, Frankfurt, Germany), after which the cell numbers was assessed. Untreated controls were defined as 100%. (B) GBM stem cells (SC) or differentiated cells (DC) were cultured for 72 hrs either in the presence or absence of 50μM U0126, followed by FACS analysis of the DNA fragmentation of propidium iodide-stained nuclei. Treatment induced DNA fragmentation, a surrogate for apoptosis induction, is shown relative to spontaneous cell death of untreated cells. (C) Shown is the relative cell viability of G35, G38 or G40 GBM stem cells after treatment with a combination of 50μM U0126 and either 100 μM temozolomide (TMZ) for 120 hrs (left) or 10nM irinotecan for 72 hrs (right). (D) Shown is the relative cell viability of G35, G38 or G40 differentiated GBM cells after treatment with a combination of 50μM U0126 and either 100 μM temozolomide (TMZ) for 120 hrs (left) or 10nM irinotecan for 72 hrs (right). (E) The relative amounts of differentiated cells (as defined by adhesion) was determined 72 hrs after initiation of differentiation, either in the presence or absence of 50μM U0126. Shown are the mean+SD of at least three independent experiment performed at least in triplicate. Red numbers in A, B and E indicate the p-value derived from a two-sided Student's t-test, the red bar in C and D indicates the statistical value that defines the mean of an additive effect. (TIF) [file pone.0131670.s005.tif]
